# Large-Scale Plant Production of *Lycium barbarum* L. by Liquid Culture in Temporary Immersion System and Possible Application to the Synthesis of Bioactive Substance

**DOI:** 10.3390/plants9070844

**Published:** 2020-07-04

**Authors:** Claudia Ruta, Giuseppe De Mastro, Simona Ancona, Anna Tagarelli, Francesca De Cillis, Carla Benelli, Maurizio Lambardi

**Affiliations:** 1 Department of Agricultural and Environmental Science, Università degli Studi di Bari Aldo Moro, via Amendola 165/a, 70125 Bari, Italy; claudia.ruta@uniba.it (C.R.); ancona.simona@gmail.com (S.A.); anna.tagarelli@uniba.it (A.T.); francescadecillis@gmail.com (F.D.C.); 2 IBE-Institute of BioEconomy, National Research Council (CNR), 50019 Sesto Fiorentino (Florence), Italy; carla.benelli@ibe.cnr.it (C.B.); maurizio.lambardi@ibe.cnr.it (M.L.)

**Keywords:** goji, Plantform^TM^ bioreactor, large-scale production, total phenols, total flavonoids

## Abstract

Goji (*Lycium barbarum* L.) has recognized nutritive and antioxidant properties and many products are commercialized for health in food market. Besides its food use, goji has been the subject of more than 2000 years of traditional Chinese medicine, using berries, root bark, and leaves. Here, the potential of the liquid culture in temporary immersion system (TIS) by using the bioreactor Plantform^TM^ was tested for the large-scale production of high-quality goji shoots and the subsequent production of total phenols and flavonoids. The three tested immersion cycles differently influenced the shoot quality in terms of proliferation and hyperhydricity. The best immersion cycle (time and frequency) was proven to have the shortest daily immersion time (6 min every 24 h) which ensured good levels of relative growth and multiplication rate, very limited onset of hyperydricity, and the longest shoots, promoting direct rooting after only 30 days of culture. In comparison with the semisolid culture, the TIS culture resulted in an increase of the total phenolic content (TPC) and in a lower value of the total flavonoid content (TFC). However, considering the higher quantity of biomass produced in the Plantform^TM^ bioreactor, the difference in terms of TFC productivity between semisolid medium and TIS liquid culture was proven to be statistically equivalent.

## 1. Introduction

Goji, known also as goji berry and wolfberry (*Lycium barbarum* L.), belonging to Solanaceae family, is a shrub widely present in arid and semi-arid regions of China, Southeastern Europe, and the Mediterranean area [1]. This crop became very popular in the last few years due to its nutritive and antioxidant properties [1] and many products are commercialized for health in the food market. Besides its food use, goji has been the subject of more than 2000 years of traditional Chinese medicine, using not only the berries, but also the root bark and the leaves. In particular, the leaves are consumed in tea infusions or as spices [1], and phytochemical investigations recently suggested them as an important source of phenols and flavonoids.

Plant tissue culture is considered a valuable technique for the production of bioactive compounds [2,3], as an alternative way to the exploit its natural habitat where, in addition, its harvesting is influenced by the plant growth stage, environmental stress, nutrition, and plant genetic [4]. However, micropropagation of plants is time consuming, labor intensive and expensive [5], and is commercially exploitable when the production cycle is made economically advantageous. Thus, in time, in order to increase the biomass production of plants and reduce production costs, some alternative systems to the traditional micropropagation in semisolid media have been developed. Among them, the liquid culture in bioreactors in temporary immersion system (TIS) has recently received large attention, providing suitable growth conditions under controlled environmental factors to obtain maximum quality and quantity of the plants [6,7]. In comparison to the culture in semisolid medium, the use of TIS bioreactors allows a higher contact of the whole tissues with the nutrient medium and plant growth regulators, as well as a better dilution of toxic compounds and aeration. Moreover, using bioreactors the cost of in vitro propagation decreases for the saving of the expansive gelling agents and a general better automation of the production cycle [8].

Permanent liquid culture in bioreactor of plant tissues and organs was the first system proposed [9] where cultures into the liquid medium are completely immersed. That condition causes, in several species, physiological disorder and loss of material due to asphyxia and hyperhydricity for the low oxygen content and the high water retention capacity [10]. TIS could overcome these difficulties by flooding of plant tissue at intermittent and short-lasting time intervals with liquid culture medium that ensures a proper supply of nutrients and gas transfers, and decreases mechanical stress [11,12]. Different TIS bioreactor types have been effectively used for the successful mass propagation of numerous plant species, which notably include *Aloe barbadensis* Mill. [13], *Olea europaea* L. [14], *Quercus robur* L. [15], and *Vanilla planifolia* Jacks. Ex Andrews [16]. In addition their successful use in the commercial micropropagation of economically important plant species [17], TIS bioreactors have been tested also in secondary metabolite production [18], molecular farming [19], and even in phytoremediation from toxic compounds [20]. Among the recently proposed TIS systems, the effectiveness of Plantform^TM^ for the large-scale micropropagation of ornamental and fruit plants has been demonstrated [8,11].

The aim of the present study was to develop and optimize a protocol for the in vitro propagation of *L. barbarum*, evaluating the potential of the TIS bioreactor Plantform^TM^ for the large-scale production of high-quality goji shoots, and the subsequent production of total phenols and flavonoids.

## 2. Results and Discussion

### 2.1. Seed Germination, Shoot Induction and Multiplication

**Figure 1:**
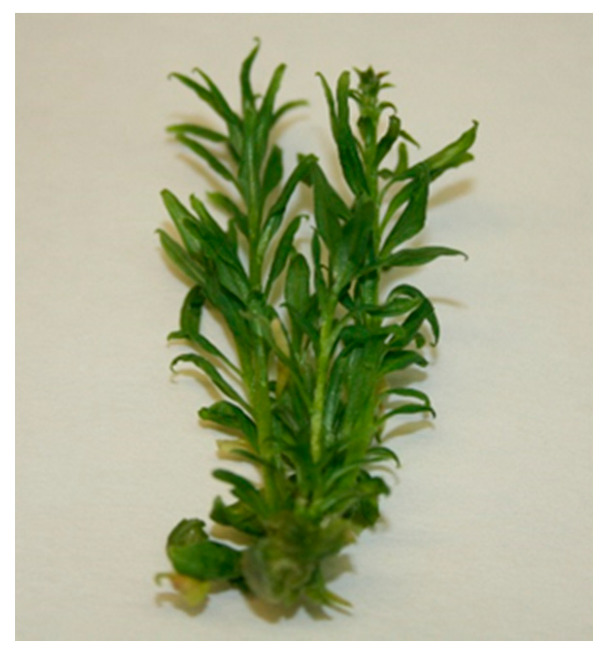
Cluster of goji shoots obtained on MB (see Material and Methods) added with 0.1 mg L^−1^ BAP after 4 weeks of culture in semisolid medium.

In vitro germination of goji seeds was easily obtained at a rate of 90%. After 3 weeks, seedlings reached 4–5 cm of height and, at that time, the explants were obtained and transferred in the proliferation medium. Among the tested concentrations of 6-benzylaminopurine (BAP), the concentration of 0.1 mg L^−1^ induced the highest average number and length of shoots per explant (4.8 and 4.6 cm, respectively; Table 1, Figure 1).

**Figure 2:**
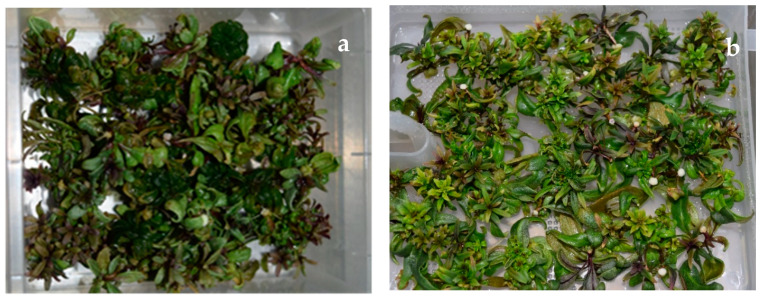
Different shoot quality in different culture conditions: (**a**) clear symptoms of hyperhydricity of the shoots when cultured in semisolid medium and Sterivent containers; (**b**) high quality of the culture in TIS bioreactor after 30 days with immersion cycle of 6 min every 24 h (P1).

A majority of reports concerning goji deal with in vitro organogenesis and somatic embryogenesis. Some authors reported the use of BAP in combination with α-napthaleneacetic acid (NAA) to induce direct or indirect organogenesis, and indirect somatic embryogenesis of goji starting from node or leaf explants. One example is the study of Osman et al. [21], where the best result of plantlet regeneration through indirect organogenesis was obtained combining BAP and NAA (both at 0.5 mg L^−1^) starting from leaf explants. Leaf explants were also identified as the better way to induce indirect somatic embryogenesis on Murashige and Skoog (MS) medium [22], added of 1.0 mg L^−1^ 2,4-dichlorophenoxyacetic acid and 0.1 mg L^−1^ BAP [23]. Adventitious shoots were, instead, directly produced from 2–3 node fragments on MS medium with different combinations of NAA and BAP [24], as well as from leaves cultured on MS medium, added of 2 mg L^−1^ BAP and 0.5 mg L^−1^ NAA [25]. One aim of the present study was to develop an effective micropropagation protocol by axillary shoot formation and shoot proliferation, avoiding the formation of calluses or adventitious budding. For this reason, BAP was tested at different concentrations without the contemporary use of auxins. The axillary buds from germinated seedlings responded promptly to the in vitro condition. No statistically significant difference was shown between the tested concentrations, both in shoot number and shoot length. Fira et al. [26], using larger microcuttings (2 cm in length, with 4–5 nodes) as explants in the micropropagation of goji berry, and testing BAP at various concentrations (from 0.44 to 8.88 μM), obtained the highest proliferation rate when the treatment with 2.22 μM was applied, while the 1.33 μM concentration provided longer and more robust shoots. The 2.22 μM concentration corresponds to the intermediate concentration used in the present study (0.5 mg L^−1^), while shoots growing on the highest concentration of BAP (1.0 mg L^−1^) showed often shoots with clear signs of hyperhydricity and anomalous clustering (Figure 2a).

Hyperhydricity is a physiological disorder typical of in vitro culture that seriously affects shoot proliferation and vitality [27]. This phenomenon is linked to various stress factors, and in particular to the culture media composition, including imbalanced or high levels of plant growth regulators [28]. Kataeva et al. [29] evaluated the natural content of cytokinins in vitrified shoots of apple. They supposed that the excess of cytokinins could be responsible for the shoot hyperhydricity by inducing rapid divisions of cells in meristems, under the high humidity of tightly closed vessels. Therefore, it can be hypothesized that the development of hyperhydricity in goji shoots was induced here by a high amount of BAP, according also to similar results in *Aloe polyphylla* [30].

### 2.2. Shoot Growth and Proliferation in Different Culture Systems

The relative growth rate (RGR) values after 30 days of culture in Plantform^TM^ bioreactors and in Sterivent containers (control) showed a significant higher biomass production in the TIS liquid culture (Table 2). No consistent difference occurred among the three tested immersion cycles.

In addition, the multiplication rate (MR) was also higher in the TIS culture, with the average value that was double in P1, in comparison to the conventional micropropagation in semisolid medium (4.4 vs. 2.2). On the contrary, the highest shoot length was obtained from the shoots grown on semisolid medium, with values significantly higher (5.5 cm), than the ones reached in P1, P3 and P2 (4.0, 2.8 and 2.5 cm, respectively). Among the three immersion cycles, 8 min every 16 h (P2) and 20 min every 12 h (P3) produced high percentages of hyperhydricity (100% and 85%, respectively). Instead, the immersion cycle of 6 min every 24 h (P1) produced healthy shoots with minimum signs of hyperhydricity (Figure 2b), with a very high percentage of rooted shoots, although in auxin-free medium (see also “Effect of the two culture systems on shoot rooting”).

Our findings show that the increment of the immersion time produced a negative effect on shoots quality, and demonstrated the success of Plantform^TM^ bioreactors for the micropropagation of goji in TIS liquid culture by increasing the total biomass and the proliferation of shoots, following the selection of a suitable immersion time, compared to the classical culture in semisolid medium. The effectiveness of this TIS bioreactor has been underlined by other Authors working with different species, i.e., *Digitalis lutea × purpurea* L. and *Echinacea purpurea* (L) Moench [11], *Olea europaea* L. [14] and *Quercus ruber* L. [15]. The liquid medium promotes a greater availability and uptake of nutrients, less variation in pH and conductivity, and attenuation of inhibiting compounds [31]. On the other hand, the length and frequency of TIS of explants are of great importance and their proper evaluation is fundamental to achieve an optimal shoot culture [32]. On the contrary, unsuitable parameters can cause hyperhydricity and asphyxia of plant tissue [9].

### 2.3. Effect of the Two Culture Systems on Shoot Rooting

As shown in Table 2, the shoots cultured in Plantform^TM^ with the immersion cycle of 6 min every 24 h (P1) achieved as much as 80% of rooting after 30 days in auxin-free BM (see “4. Material and Methods”) liquid medium. Instead, only 5% of rooted shoots was obtained on the same semisolid medium in Sterivent containers.

In order to induce higher shoot rooting in semisolid medium, the transfer of shoots from Sterivent containers to fresh semisolid medium, with or without indole-3-butyric acid (IBA) addiction, was necessary. After only one week from the transfer, the shoots presented root-tip emergence, and 50% of rooted shoots was achieved even in hormone-free medium, with an average root number that was higher (5.9) to the ones from IBA-treated shoots (Table 3). At the end of the third week, the shoots which had been treated with 0.3 mg L^−1^ of IBA achieved a rooting of 82%, with no significant differences from the others tested IBA concentrations (78% and 80% for 0.5 and 1.0 mg L^−1^, respectively). The average length of adventitious roots was equivalent in all the IBA-treated and not treated shoots (from 1.4 to 1.6 cm).

This study confirms the results of other reports dealing with *L. barbarum*, where it was evidenced the possibility to induce shoot rooting with or without auxin treatment [24,25]. However, the most interesting outcome is the possibility to induce, at the same time, shoot proliferation and rooting in TIS liquid culture by using the Plantform^TM^ bioreactor, hence reducing by two weeks the time required to obtain at least 80% of rooted plantlets ready to be transferred in acclimatization.

### 2.4. Determination of Total Phenolic and Flavonoid Contents

The evaluation of total phenolic content (TPC) and total flavonoid content (TFC) in goji in vitro culture was determined on shoots obtained from Plantform^TM^, at the shortest immersion time per day (P1), and semisolid medium. The TIS liquid culture resulted in increasing the TPC, as determined as mg GAE g^−1^ dry weight of the goji shoots (Table 4). Indeed, the parameters detected from Plantform^TM^ were 3.9 g, 23.6 mg GAE g^−1^ dry weight, and 92.6 mg per bioreactor, all significantly higher than the values detected from shoots cultured in semisolid medium. On the contrary, shoots grown on semisolid medium showed a significant higher value in TFC in comparison with that extracted from the shoots grown in TIS (18.5 mg RE g^−1^ dw vs. 10.9 mg RE g^−1^ dw). However, considering the higher quantity of biomass produced in Plantform^TM^ bioreactor, the difference in terms of TFC productivity between semisolid medium and TIS liquid culture resulted to be statistically equivalent (51.8 mg vs. 42.5 mg).

Even if the health benefices of goji leaves are well recognized in traditional Chinese medicine and in many Asian countries, nowadays, the interest is increasing also in other countries for their potential uses in foods or health-promoting formulations as a rich source of bioactive compounds [33]. In particular, among the phytochemicals, phenolic and flavonoid compounds are the most interesting and investigated [1,33]. In this context, tissue culture is a valuable technique to produce secondary metabolites, and many studies showed the improvements that can be obtained in many species such as, in the same Lamiaceae family, sage [3] and oregano [34]. The advantage to produce antioxidants by goji in vitro culture was also underlined by Osman et al. [21] and Dutu et al. [35] on 2-month old plantlets, using the conventional micropropagation. In particular, the latter Authors tested the influence of different lengths wave (white; blue 473 nm; green 533 nm; yellow 580 nm; red 680 nm) on TPC and TFC content of goji leaves. The leaves were cut from plantlets grown for 60 days at the temperature of 23 °C, under light (16 h), and 20 °C during darkness (8 h) on MS enriched with 0.5 mg L^−1^ IBA and 0.5 mg L^−1^ BAP. In comparison with the best TPC content reached under the yellow light (1.3 mg g^−1^ of fresh weight), in the present study, a slightly higher content was obtained after 4 weeks of TIS liquid culture, i.e., 1.6 mg g^−1^ of fresh weight (data not shown). No comparison was possible between the two studies in terms of TFC, as the parameter was expressed in different ways (quercetin equivalent and rutin equivalent).

In a study on the content of bioactive compound in leaves [33], collected in vivo during the summer from wild and cultivated goji, the TPC values were 11.14 and 11.98 mg GAE g^−1^ of dw, respectively. These contents were largely lower than the quantity measured from the in vitro leaves of the present study, both in semisolid medium, and in TIS (19.4 and 23.6 mg GAE g^−1^ of dry weight respectively). Moreover, the results of another similar in vivo study [1] on flavonoid leaves contents of cultivated and wild *L. barbarum* showed values of 16.03–16.33 mg RE g^−1^ dw for the cultivated goji, little lowers than the mean content obtained in the leaves from the semisolid medium of the present study (18.5 mg RE g^−1^ dw). In the same report, the leaves from spontaneously growing plants of goji showed a lower flavonoid amount in comparison to goji leaves from TIS (6.24–7.55 and 10.9 mg RE g^−1^ dw, respectively).

## 3. Conclusions

To our knowledge, this is the first time that the liquid culture in TIS by using the Plantform^TM^ bioreactor was evaluated for the large-scale production of high-quality shoots and bioactive compounds of goji (*L. barbatum*). In summary, the three immersion frequencies tested in this study differently influenced the shoot quality in terms of proliferation and hyperhydricity. The definition of the best immersion cycle (time and frequency), P1, i.e., the one with the shortest daily immersion time (6 min every 24 h), ensured good levels of RGR and MR, very limited onset of hyperydricity, and the longest shoots, promoting direct rooting after only 30 days of culture, and allowing for the possibility to have material to be maintained in proliferation or directly transferred in acclimatization. Furthermore, the production of biomass and bioactive compounds from in vitro goji by TIS bioreactors represents a potential for scaled-up studies on a commercial automated micropropagation system, thus being economically competitive to obtain high-value secondary metabolites.

## 4. Material and Methods

### 4.1. Plant Material and Explant Establishment

Seeds extracted from ripe berries of *L. barbarum* (GOJI ITALIANO^®^) were sterilized with 0.1% HgCl_2_ solution (*w*/*v*) for 10 min under the sterile air of a laminar flow hood. Then seeds were inoculated into 30-cc glass tubes on a basal medium (BM), consisting of MS macroelements, Nitsch and Nitsch microelements [36], 40 mg L^−1^ Fe-EDTA, 0.4 mg L^−1^ thiamine HCl, 100 mg L^−1^ myo-inositol and 7 g L^−1^ agar, without sucrose and hormone free. pH was adjusted to 5.6–5.8 before autoclaving at 121 °C for 20 min.

### 4.2. Culture Establishment and Multiple Shoot Induction

Axillary buds were excised from germinated seedlings and were cultured for shoot induction and proliferation on BM medium, supplemented with BAP at 0.1, 0.5 or 1.0 mg L^−1^, and 20 g L^−1^ sucrose into glass vessels (200 mL). Induced shoots were then divided and subcultured to fresh medium three times, each time for 4 weeks. Data concerning in vitro proliferation were recorded after each subculture, evaluating (i) the mean number of shoots per explant, and (ii) the mean length of elongated shoots. The experiment was repeated twice, with 10 vessels per replication, each containing 10 explants.

### 4.3. Shoot Proliferation and Biomass Production In Semisolid and TIS Culture Systems

**Figure 3:**
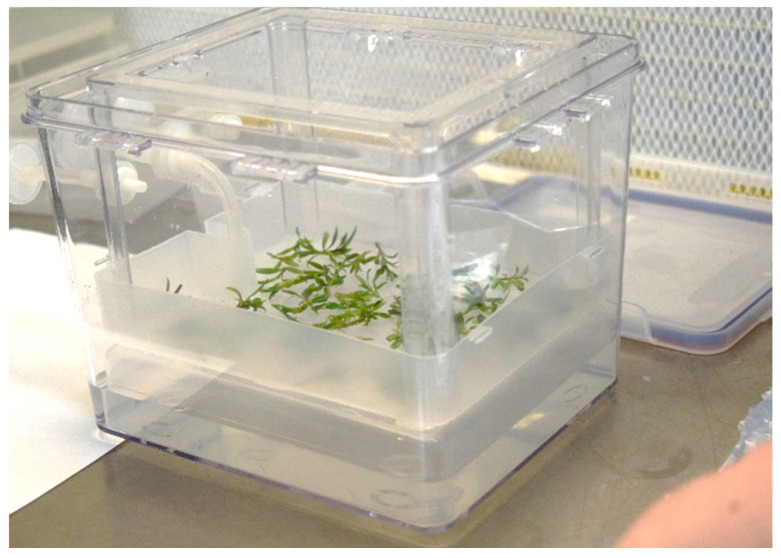
Microcuttings of goji transferred into plantform.

After the 3 subcultures of shoot induction and establishment, the shoots obtained with the selected BAP concentration were tested for the further proliferation in 2 different culture systems: in Sterivent containers (in gelled semisolid medium) and in TIS liquid culture in Plantform^TM^ bioreactors. Shoot proliferation was evaluated starting from 1.0–1.5 cm long microcuttings (Figure 3).

Sterivent containers (DUCHEFA BIOCHEMIE B.V, Haarlem, The Netherlands) are polypropylene containers of rectangular shape (107 × 94 × 96 mm), having a labyrinth closure that guarantees a continuous ventilation with the outer atmosphere, without the risks of contamination. Plantform^TM^ bioreactors *(*http://www.plantform.se/pub/*,* Sweden) are polypropylene containers of rectangular shape (180 × 160 × 150 mm) with a basket inside where the material is positioned. Two electronic timers control two distinct air pumps, regulating the frequency and immersion time first, and the ventilation cycle second [11].

In order to optimize the daily cycle of immersion, 3 different immersion cycles were tested: (i) 6 min of immersion every 24 h (P1), (ii) 8 min every 16 h (P2, corresponding to 12 min every day), and (iii) 20 min every 12 h (P3, corresponding to 40 min every day). Ventilation inside the boxes was adjusted to 15 min every 4 h. The BM culture medium, added of 0.1 mg L^−1^ BAP (as selected from the previous trial), was used to compare the two culture systems for multiple shoot induction. Before their use, bioreactors were autoclaved and the culture medium (0.5 L per container) was dispensed under the sterile air flow of the laminar flow hood. Three Plantform^TM^ for each immersion cycle, each containing 60 explants, were used in the trial.

Five Sterivent containers, each containing 12 explants, were set up for each Plantform^TM^ bioreactor, in order to compare the same number of explants (60). Hence, 180 explants were used for each culture system (Plantform^TM^ or Sterivent) and each TIS immersion cycle (3 Plantform^TM^ × 60 explants, or 15 Sterivent × 12 explants = 180 explants), with a total of 540 explants cultured in Plantform^TM^ and 540 in Sterivent, in each trial. All the containers were maintained at 23 ± 1 °C, under continuous daylight fluorescent tubes with light intensity of 50 µmol s^−1^ m^−2^ and 16 h photoperiod.

Data were collected after 30 days of culture and, for each container, consisted of (i) the multiplication rate (MR), (ii) the percentage of hyperidric shoots, (iii) the average length of shoots, and (iv) the percentage of rooted shoots. Moreover, in order to compare the two culture systems in terms of culture productivity, the RGR index [37] was used, calculated as:

RGR = (ln FW_final_ – ln FW_initial_) × 100/days of culture (1)

where ln is natural logarithm, FW_initial_ and FW_final_ are the fresh weights determined at the beginning and end of the culture period, respectively.

### 4.4. In Vitro Rooting Induction

As a large percentage of shoots directly rooted in the Plantform^TM^ bioreactors (see “*2.3. Effect of the Two Culture Systems on Shoot Rooting*”), a rooting trial was carried out only for the shoots coming from the Sterivent containers. Hence, elongated shoots, proliferated in semisolid medium, were transferred on BM medium, containing 0.0, 0.3, 0.5, or 1.0 mg L^−1^ of IBA for root induction. The percentage of rooted shoots, the mean number, and the mean length of roots per rooted shoot were then evaluated after 3 weeks. The experiment was carried out in two replications with 10 glass vessels (200 mL) per replication, each containing 10 explants. All the vessels were maintained at the conditions described above.

### 4.5. Determination of Total Phenolic Content

The TPC was measured using the Folin-Ciocalteu method, with some modification, according to the method described by Mocan et al. [33]. In short, the leaves of each sample (consisting of a pool of leaves from each container, i.e., TIS bioreactor or Sterivent) were dried and reduced to powder up to 300 microns. Then 1 g of sample was weighted in a test tube and extracted with 10 mL of a 70% ethanol solution in an ultrasonic bath at 60 °C for 30 min. The samples were cooled and centrifuged at 4500 rpm for 15 min. Afterwards, 2 mL from each ethanolic extract was diluted 25 times with the same solution, then 50 µL was mixed with 1 mL of Folin-Ciocalteu reagent, 10 mL of distilled water and diluted to 25 mL with solution of sodium carbonate (at a concentration of 290 g L^−1^). The samples were incubated in the dark for 30 min. The absorbance was measured at 760 nm, using a T60U spectrophotometer (PG Instruments, UK). TPC was expressed as mg gallic acid g^−1^ dry plant material (mg GAE g^−1^ plant material) obtained by a calibration with a standard of gallic acid at different concentrations.

### 4.6. Determination of Total Flavonoid Content

The total flavonoids content (TFC) was measured on the same extract prepared for TPC analysis. TFC was expressed as rutin equivalents (RE) and determined according to the method reported by Dong et al. [1]. Each extract (5 mL) was mixed with sodium acetate (5.0 mL, 100 g L^−1^), aluminum chloride (3.0 mL, 25 g L^−1^), and made up to 25 mL in a calibrated flask with methanol. Each solution was measured at 430 nm with the spectrophotometer (T60U; PG Instruments, UK) and compared with the same mixture without reagent (as blank). The TFC values were determined using rutin as the calibration standard [1].

### 4.7. Statistical Analysis

Each experiment was repeated twice. Data were subjected to analysis of variance (ANOVA), using the CoStat software. The Student–Newman–Keuls (SNK) test (*p* ≤ 0.05) was used to compare the means of the different treatments. Before using in the ANOVA, percentage data were subjected to the angular transformation.

## Figures and Tables

**Table 1:** Effect of different concentrations of BAP on the mean number and length of shoots, as average of 3 subsequent subcultures of 4 weeks each one.

BAP (mg L^−1^)	Shoots (n.)	Shoots Length (cm)
0.1	4.8 ^a^	4.6 ^a^
0.5	4.4 ^a^	4.5 ^a^
1.0	4.2 ^a^	3.9 ^a^

In each column, different letters indicate significantly different. Values at *p* ≤ 0.05 (SNK test). BAP, 6-benzylaminopurine, n., number.

**Table 2:** Effect of different immersion cycles on the growth (RGR), multiplication rate, hyperidricity and root production of *L. barbarum* shoots, cultured in Plantform^TM^ for 30 days.

Treatment	Shoot Proliferation and Rooting				
Immersion Cycle	RGR Index	MR (n)	Hyperidricity (%)	Length (cm)	Rooting (%)
P1	8.79	4.4 ^a^	10 ^b^	4.0 ^b^	80 ^a^
P2	9.35	4.2 ^a^	85 ^a^	2.8 ^c^	35 ^b^
P3	7.91	4.1 ^a^	100 ^a^	2.5 ^c^	0 ^c^
Control	6.91	2.2 ^b^	5 ^b^	5.5 ^a^	5 ^c^

In each column, different letters indicate significantly different values at *p* ≤ 0.05 (SNK test). MMR, multiplication rate; RGR, relative growth rate; Control, shoot culture in semisolid medium. P1, 6 min of immersion every 24 h; P2, 8 min every 16 h; P3, 20 min every 12 h.

**Table 3:** Effect of different concentrations of IBA on rooting percentage, average number and average length of roots of *L. barbarum* shoots, recorded after 3 weeks from transfer on semisolid rooting medium.

IBA (mg L^−1^)	Rooting (%)	Root Number (n.)	Root Length (cm)
0.0	50 ^b^	5.9 ^a^	1.4 ^a^
0.3	82 ^a^	4.8 ^a^	1.6 ^a^
0.5	78 ^a^	3.9 ^a^	1.4 ^a^
1.0	80 ^a^	3.2 ^a^	1.5 ^a^

In each column, with different letters indicating significant differences at *p* ≤ 0.05 (SNK test).

**Table 4:** Effect of different culture systems on bioactive compound accumulation, measured in goji shoots after 4 weeks of culture.

		TPC		TFC	
Culture System	Biomass/Container	Content	Content/Container	Content	Content/Container
	g	mg GAE g^−1^ dw	mg	mg RE g^−1^ dw	mg
Plantform^TM^ (P1)	3.9 ^a^	23.6 ^a^	92.6 ^a^	10.9 ^b^	42.5 ^a^
Semisolid medium	2.8 ^b^	19.4 ^b^	54.8 ^b^	18.5 ^a^	51.8 ^a^

In each column, with different letters indicating significant differences at *p* ≤ 0.05 (SNK test). TPC, total phenol content; TFC, total flavonoid content; GAE, Gallic acid equivalent; RE, Rutin equivalent; dw, dry weight; P1, immersion cycle of 6 min every 24 h.

## References

[1] Dong J.Z., Lu D.Y., Wang Y. (2009). Analysis of Flavonoids from Leaves of Cultivated *Lycium barbarum* L.. Plant Foods Hum. Nutr..

[2] Murthy H.N., Lee E.J., Paek K.Y. (2014). Production of secondary metabolites from cell and organ cultures: Strategies and approaches for biomass improvement and metabolite accumulation. Plant Cell Tissue Organ Cult..

[3] Savona M., Barberini S., Bassolino L., Mozzanini E., Pistelli L., Pistelli L., Ruffoni B., Georgiev V., Pavlov A. (2017). Strategies for Optimization of the Production of Rosmarinic Acid in *Salvia officinalis* L. and *Salvia dolomitica* Codd Biomass with Several Biotechnological Approaches. Salvia Biotechnology.

[4] Debnath S.C. (2017). Temporary immersion and stationary bioreactors for mass propagation of true-to-type highbush, half-high, and hybrid blueberries (*Vaccinium* spp.). J. Hortic. Sci. Biotechnol..

[5] Ahmadian M., Babaei A., Shokri S., Hessami S. (2017). Micropropagation of carnation (*Dianthus caryophyllus* L.) in liquid medium by temporary immersion bioreactor in comparison with solid culture. J. Genet. Eng. Biotechnol..

[6] Georgiev V., Schumann A., Pavlov A., Bley T. (2014). Temporary immersion systems in plant biotechnology. Eng. Life Sci..

[7] Carvalho L.S.O., Ozudogru E.A., Lambardi M., Paiva L.V. (2019). Temporary Immersion System for Micropropagation of Tree Species: A Bibliographic and Systematic Review. Not. Bot. Horti Agrobo..

[8] Lambardi M. (2012). Micropropagazione in coltura liquida con sistema ad immersione temporanea. Riv. Fruttic. Ortofloric..

[9] Ziv M., Hvoslef-Eide A., Preil W. (2005). Simple bioreactors for mass propagation of plants. Liquid Culture Systems for In Vitro Plant Propagation.

[10] Kevers C., Franck T., Strasser R.J., Dommes J., Gaspar T. (2004). Hyperhydricity of micropropagated shoots: A typically stress-induced change of physiological state. Plant Cell Tissue Organ Cult..

[11] Welander M., Persson J., Asp H., Zhu L.H. (2014). Evaluation of a new vessel system based on temporary immersion system for micropropagation. Sci. Hortic..

[12] Mosqueda Frometa O., Escalona Morgado M.M., Da Silva J.A.T., Pina Morgado D.T., Daquinta Gradaille M.A. (2017). In vitro propagation of *Gerbera jamesonii* Bolus ex Hooker f. In a temporary immersion bioreactor. Plant Cell Tissue Organ Cult..

[13] Cardarelli M., Cardona Suarez M.C., Colla G. (2014). Influence of ozone treatments on in vitro propagation of *Aloe barbadensis* in continuous immersion bioreactor. Ind. Crops Prod..

[14] Benelli C., De Carlo A. (2018). In vitro multiplication and growth improvement of *Olea europaea* L. cv Canino with temporary immersion system (Plantform™). 3 Biotech.

[15] Gatti E., Sgarbi E., Ozudogru E.A., Lambardi M. (2017). The effect of PlantformTM bioreactor on micropropagation of *Quercus robur* in comparison to a conventional in vitro culture system on gelled medium, and assessment of the microenvironment influence on leaf structure. Plant Biosyst. Int. J. Deal. All Asp. Plant Biol..

[16] Spinoso-Castillo J.L., Chavez-Santoscoy R.A., Bogdanchikova N., Perez-Sato J.A., Morales-Ramos V., Bello-Bello J.J. (2017). Antimicrobial and hormetic effects of silver nanoparticles on in vitro regeneration of vanilla (*Vanilla planifolia* Jacks. Ex Andrews) using a temporary immersion system. Plant Cell Tissue Organ Cult..

[17] Bello-Bello J., Canto-Flick A., Balam-Uc E., Robert L. (2010). Improvement of in vitro proliferation and elongation of Habanero pepper shoots (*Capsicum chinense* Jacq.) by temporary immersion. HortScience.

[18] Perez-Alonso N., Wilken D., Gerth A., Jahn A., Nitzsche H.-M., Kerns G., Capote-Perez A., Jiménez E. (2009). Cardiotonic glycosides from biomass of *Digitalis purpurea* L. cultured in temporary immersion systems. Plant Cell Tissue Organ Cult..

[19] Huang T.-K., McDonald K.A. (2012). Bioreactor systems for in vitro production of foreign proteins using plant cell cultures. Biotechnol. Adv..

[20] Rodrıguez-Couto S. (2011). Production of laccase and decolouration of the textile dye Remazol Brilliant Blue R in temporary immersion bioreactors. J. Hazard. Mater..

[21] Osman N.I., Awal A., Sidik N.J., Abdullah S. (2013). In Vitro Regeneration and Antioxidant Properties of *Lycium barbarum* L. (Goji). J. Teknol..

[22] Murashige T., Skoog F. (1962). A revised medium for rapid growth and bio assays with tobacco tissue cultures. Physiol. Plant.

[23] Osman N.I., Awal A., Sidik N.J., Abdullah S. (2013). Callus Induction and Somatic Embryogenesis from Leaf and Nodal Explants of *Lycium barbarum* L. (Goji). Biotechnology.

[24] Dănăilă-Guidea S.-M., Dobrinoiu R.-V., Vişan L., Toma R.C. (2015). Protocol for efficient in vitro multiplication of *Lycium barbarum* L. (goji) by direct organogenesis. Sci. Bull. Ser. F Biotechnol..

[25] Hu Z., Guo G.-Q., Zhao D.-L., Li L.-H., Zheng G.-C. (2001). Shoot Regeneration from Cultured Leaf Explants of *Lycium barbarum* and *Agrobacterium* Mediated Transformation. Russ. J. Plant Physiol..

[26] Fira A., Clapa D. (2011). Results Regarding in Vitro Proliferation in Goji (*Lycium barbarum*). Bull. UASVM Hortic..

[27] Van Den Dries N., Giannì S., Czerednik A., Krens F.A., de Klerk G.-J.M. (2013). Flooding of the apoplast is a key factor in the development of hyperhydricity. J. Exp. Bot..

[28] Hazarika B.N. (2006). Morpho-physiological disorders in in vitro culture of plants. Sci. Hortic..

[29] Kataeva N.V., Alexanandrova I.G., Butenko R.S., Dragavtcera E.V. (1991). Effect of applied and internal hormones on vitrification and apical necrosis of different plants cultured in vitro. Plant Cell Tissue Organ Cult..

[30] Ivanova M., Van Staden J. (2011). Influence of gelling agent and cytokinins on the control of hyperhydricity in *Aloe polyphylla*. Plant Cell Tissue Organ Cult..

[31] Debergh P.C., De Riek J., Matthys D., Lumsden P.J., Nicholas J.R., Davies W.J. (1994). Nutrient supply and growth of plants in culture. Physiology, Growth and Development of Plants in Culture.

[32] Etienne H., Berthouly M. (2002). Temporary immersion systems in plant micropropagation. Plant Cell Tissue Organ Cult..

[33] Mocan A., Zengin G., Sirmigiotis M., Schafberg M., Mollica A., Vodnar D.C., Crișan G., Rohn S. (2017). Functional constituents of wild and cultivated Goji (*L. barbarum* L.) leaves: Phytochemical characterization, biological profile, and computational studies. J. Enzym. Inhib. Med. Chem..

[34] Lattanzio V., Cardinali A., Ruta C., Morone Fortunato I., Lattanzio V.M.T., Linsalata V., Cicco N. (2009). Relationship of secondary metabolism to growth in oregano (*Origanum vulgare* L.) shoot cultures under nutritional stress. Environ. Exp. Bot..

[35] Duțu M., Ardelean A., Ardelean M., Cachiţă-Cosma D., Burducea M., Lobiuc A., Rosenh E. (2016). Increasing the antioxidant activity, total phenolic and assimilatory pigments content by optimizing the in vitro growth conditions of *Lycium barbarum* plant. Sci. Bull. Ser. F Biotechnol..

[36] Nitsch J.P., Nitsch C. (1969). Haploid Plants from Pollen Grains. Science.

[37] Hoffman W., Poorter H. (2002). Avoiding bias in calculations of relative growth rate. Ann. Bot..

